# Draft Genome Sequence of Rhodococcus qingshengii Strain PN_19, Isolated from a Moribund Individual of Pinna nobilis in Sardinia, Italy

**DOI:** 10.1128/MRA.00356-21

**Published:** 2021-06-10

**Authors:** Fabio Scarpa, Daria Sanna, Ilenia Azzena, Piero Cossu, Davide Mugetti, Francesco Cerutti, Marino Prearo, Simone Peletto, Marco Casu

**Affiliations:** a Department of Veterinary Medicine, University of Sassari, Sassari, Italy; b Department of Biomedical Sciences, University of Sassari, Sassari, Italy; c Istituto Zooprofilattico Sperimentale del Piemonte, Liguria e Valle d’Aosta, Turin, Italy; University of Southern California

## Abstract

During an epidemiological survey that aimed to discover the causes for the mass mortality of Pinna nobilis, a strain of *Rhodococcus* was found in a moribund individual. Here, we report its 7,037,134-bp draft genome sequence, which, after the annotation and genome survey, was identified as belonging to Rhodococcus qingshengii PN_19.

## ANNOUNCEMENT

The fan mussel, Pinna nobilis Linnaeus, 1758 (Mollusca: Bivalvia) (1, 2), is currently facing dramatic mass mortality events (MMEs), whose causes are far from being completely elucidated (3). A recent molecular survey searching for the etiological agents potentially involved in the MMEs found a strain of *Rhodococcus* in a moribund *P. nobilis* individual (4). Here, we report the draft genome sequence of that strain, labeled PN19 by Scarpa et al. (4). Isolation was performed using 100 μl of hemolymph to seed Columbia blood agar plates. After incubation for 72 h at 22 ± 2°C, monomorphic colonies were visualized and preliminarily identified by matrix-assisted laser desorption ionization–time of flight mass spectrometry (MALDI-TOF MS) as Rhodococcus erythropolis (4). A further subculture was carried out before purifying the DNA. The total number of colonies was not quantified because it was not the study goal. Genomic DNA was extracted from a single colony by boiling and freeze‐thawing. The genome was sequenced by whole-genome shotgun (WGS) sequencing on an Illumina MiSeq platform by the Istituto Zooprofilattico Sperimentale del Piemonte, Liguria e Valle d’Aosta (Turin, Italy). The DNA was processed using the Nextera XT DNA library preparation kit (Illumina), following the manufacturer’s instructions. The final library was sequenced on an Illumina MiSeq platform with the MiSeq reagent kit v3-600, in a 300-bp single-end run. The resulting FASTQ file contained 19,772,043 reads (≃100× coverage) with an average length of 274 nucleotides (nt) (maximum length, 301 nt). FASTX-Toolkit v0.7 (5) was used to remove the sequencing adapters (command line: fastx_clipper -e 0.1 -a CTGTCTCTTATACACATCT -l 15) and trim the short and low-quality sequences (command line: fastq_quality_filter -v -m 30 -Q 33 -q 25 -p 50). *De novo* assembly was performed using SPAdes v3.12.0 (6) (command line: spades.py –threads 8 -o . –data set spades_yaml_file.txt - m 128), obtaining 205 contigs of ≥300 nt (for a total of 212), with the largest one being 854,599 nt. The *N*_50_ length (the shortest sequence length at 50% of the genome) is 220,785 bp, while the *L*_50_ count (the smallest number of contigs whose length sum produces *N*_50_) amounts to 9. The *N*_75_ and *L*_75_ values are 74,147 bp and 25 contigs, respectively. The estimated genome length is 7,037,134 nt, with an average G+C content of 62.49%.

The systematic rank of the annotated genome, verified using PATRIC, the Bacterial Bioinformatics Resource Center (https://www.patricbrc.org/) (7), is as follows: cellular organisms, *Bacteria* (*Terrabacteria* group), *Actinobacteria, Corynebacteriales, Nocardiaceae, Rhodococcus*, Rhodococcus qingshengii.

The whole draft genome was annotated using the NCBI Prokaryotic Genome Annotation Pipeline (PGAP) (8) with the best-placed reference protein set method and GeneMarkS-2+. Information about PGAP can be found at https://www.ncbi.nlm.nih.gov/genome/annotation_prok/.

The annotated genome contains 6,656 predicted protein-coding genes (CDS), 53 tRNAs, and 5 rRNA genes.

### Data availability.

The whole-genome shotgun sequence of Rhodococcus qingshengii PN_19 described here has been deposited in NCBI GenBank, under the following codes: BioProject accession number PRJNA717236 (Taxonomy accession number 334542), BioSample accession number SAMN18490894 (Taxonomy accession number 1833), and GenBank accession number JAGIXV000000000 (JAGIXV010000001.1 through JAGIXV010000212). The raw sequence reads used for assembly have been deposited in the Sequence Read Archive (SRA) under accession number SRR14301821. The version described in this paper is JAGIXV010000000.
